# Genome-wide screen reveals cellular functions that counteract rifampicin lethality in *Escherichia coli*

**DOI:** 10.1128/spectrum.02895-23

**Published:** 2023-12-06

**Authors:** Yu Wang, Han Fu, Xiao-Jie Shi, Guo-Ping Zhao, Liang-Dong Lyu

**Affiliations:** 1 Key Laboratory of Medical Molecular Virology of the Ministry of Education/National Health Commission, School of Basic Medical Sciences and Department of Microbiology and Microbial Engineering, School of Life Sciences, Fudan University, Shanghai, China; 2 CAS Key Laboratory of Synthetic Biology, CAS Center for Excellence in Molecular Plant Sciences, Chinese Academy of Sciences (CAS), Shanghai, China; 3 University of Chinese Academy of Sciences, Beijing, China; 4 Shanghai Key Laboratory of Tuberculosis, Shanghai Clinical Research Center for Infectious Disease (Tuberculosis), Shanghai Pulmonary Hospital, Shanghai, China; The University of North Carolina at Chapel Hill, Chapel Hill, North Carolina, USA

**Keywords:** rifamycins, antibiotic susceptibility, drug resistance, Tn-Seq

## Abstract

The antibacterial activity of rifamycins specifically relies on the inhibition of transcription by directly binding to the β-subunit of bacterial DNA-dependent RNA polymerase (RNAP). However, its killing efficacy is substantially diminished in most gram-negative bacteria. To systematically reveal the cellular functions that counteract rifamycin-mediated killing in the gram-negative model organism *Escherichia coli*, we performed a genome-wide Tn5 transposon-mediated screen to identify mutants with altered susceptibility to rifampicin. Combined with targeted gene knockouts, our results showed that the β-barrel assembly machinery plays a crucial role in restricting rifampicin from entering the cell, whereas mutants deficient in other cellular permeability barriers, such as lipopolysaccharide and enterobacterial common antigen, had no such effect. At bactericidal concentrations, the killing efficacy of rifampicin was strongly influenced by cellular functions, including iron acquisition, DNA repair, aerobic respiration, and carbon metabolism. Although iron acquisition *de facto* has a strong impact or dependence on cellular redox, our results suggest that their effects on rifampicin efficacy do not rely on hydroxyl radical formation. We provide evidence that maintenance of DNA replication and transcription-coupled nucleotide excision repair protects *E. coli* cells against rifampicin killing. Moreover, our results showed that sustained aerobic respiration and carbon catabolism diminish rifampicin’s killing efficacy, and this effect relies on the inhibition of transcription but not on translation. These findings suggest that the killing efficacy of rifamycins is largely determined by cellular responses upon the inhibition of RNAP and may expand our knowledge of the action mechanisms of rifamycins.

**IMPORTANCE:**

Rifamycins are a group of antibiotics with a wide antibacterial spectrum. Although the binding target of rifamycin has been well characterized, the mechanisms underlying the discrepant killing efficacy between gram-negative and gram-positive bacteria remain poorly understood. Using a high-throughput screen combined with targeted gene knockouts in the gram-negative model organism *Escherichia coli*, we established that rifampicin efficacy is strongly dependent on several cellular pathways, including iron acquisition, DNA repair, aerobic respiration, and carbon metabolism. In addition, we provide evidence that these pathways modulate rifampicin efficacy in a manner distinct from redox-related killing. Our findings provide insights into the mechanism of rifamycin efficacy and may aid in the development of new antimicrobial adjuvants.

## INTRODUCTION

Rifamycins comprise a group of natural or semi-synthetic antibiotics with a wide antibacterial spectrum (1, 2). Although it is well established that the action of rifamycins depends on the inhibition of transcription by directly binding to the β-subunit of bacterial DNA-dependent RNA polymerase (RNAP) (3), their killing efficacy on various organisms differs profoundly. For instance, rifampicin (a semi-synthetic derivative of rifamycins) is a cornerstone of short-course antituberculosis therapy because of its sterilizing activity against both replicating and nonreplicating antibiotic-tolerant *Mycobacterium tuberculosis*. This sterilizing activity is barely observed in most bacteria that are *de facto* sensitive to rifampicin (4).

Early investigations attributed this difference in antimicrobial activity to the efficacy of drug uptake, as most gram-negative bacteria show lower susceptibility to rifamycins owing to the presence of the outer membrane (OM) (2). However, the penetration of the antibiotic through the cell membrane may account for rifamycin susceptibility in terms of minimum inhibitory concentration (MIC) but not for killing efficacy, especially when considering that rifamycins do not exhibit a typical concentration-dependent killing effect on *Escherichia coli* (2). In fact, even at concentrations that could completely inhibit RNAP, the killing efficacy of rifampicin remains mild in *E. coli* (5 – 9). Moreover, biochemical studies have demonstrated that the difference in rifampicin’s killing efficacy is unlikely to stem from variations in their binding site on RNAP, as RNAPs from both gram-negative and gram-positive bacteria have comparable sensitivities to rifampicin (9, 10). These results indicate that rifamycin lethality may depend on cellular responses to RNAP inhibition (11, 12).

Mounting evidence indicates that bacterial metabolic states and responses have a strong impact on antibiotic efficacy (12, 13). The immediate downstream events of RNAP inhibition are the prevention of transcription and translation, both of which are major energy-consuming processes of cellular activities (7, 14, 15). Previous studies have shown that blocking RNA synthesis by rifampicin leads to a reduced rate of aerobic respiration and metabolism (5). Although these cellular responses could protect bacteria from bactericidal antibiotics, their role in rifampicin’s efficacy remains unclear.

In bacteria, the cellular response to DNA damage has been associated with antibiotic efficacy, as well as other lethal stresses (16 – 19). In *E. coli*, the deletion of *recA* results in hypersensitivity to rifampicin killing (6). In mycobacteria, independent lines of evidence suggested a role of reactive oxygen species (ROS) in rifampicin-induced lethal DNA damage (17, 20 – 23). However, because rifampicin inhibits aerobic respiration and does not stimulate ROS production in *E. coli* (5, 24), the molecular mechanisms underlying DNA damage and damage repair upon rifamycin action remain elusive.

To systematically reveal the cellular functions that influence rifamycin lethality, we performed genome-wide screening of the *E. coli K12* strain MG1655. Through the construction of a high-density Tn5 transposon mutation library (71.4/kb) and deploying high-throughput transposon insertion sequencing (Tn-Seq) (25), we comprehensively identified the mutants with altered fitness upon exposure to different rifampicin concentrations (0.25× MIC to 20× MIC). Combined with targeted gene knockouts, our results demonstrated that the efficacy of rifampicin strongly relies on cellular functions, including iron acquisition, DNA repair, aerobic respiration, and carbon metabolism. Further, we provided evidence that these pathways modulate rifampicin’s efficacy in a manner that is distinct from redox-related killing. These findings provide insights into the mechanism of action of rifamycins and may aid in the development of new antimicrobial strategies to improve rifamycin efficacy.

## RESULTS

### Selection of transposon mutants altering rifampicin efficacy

**Fig 1:**
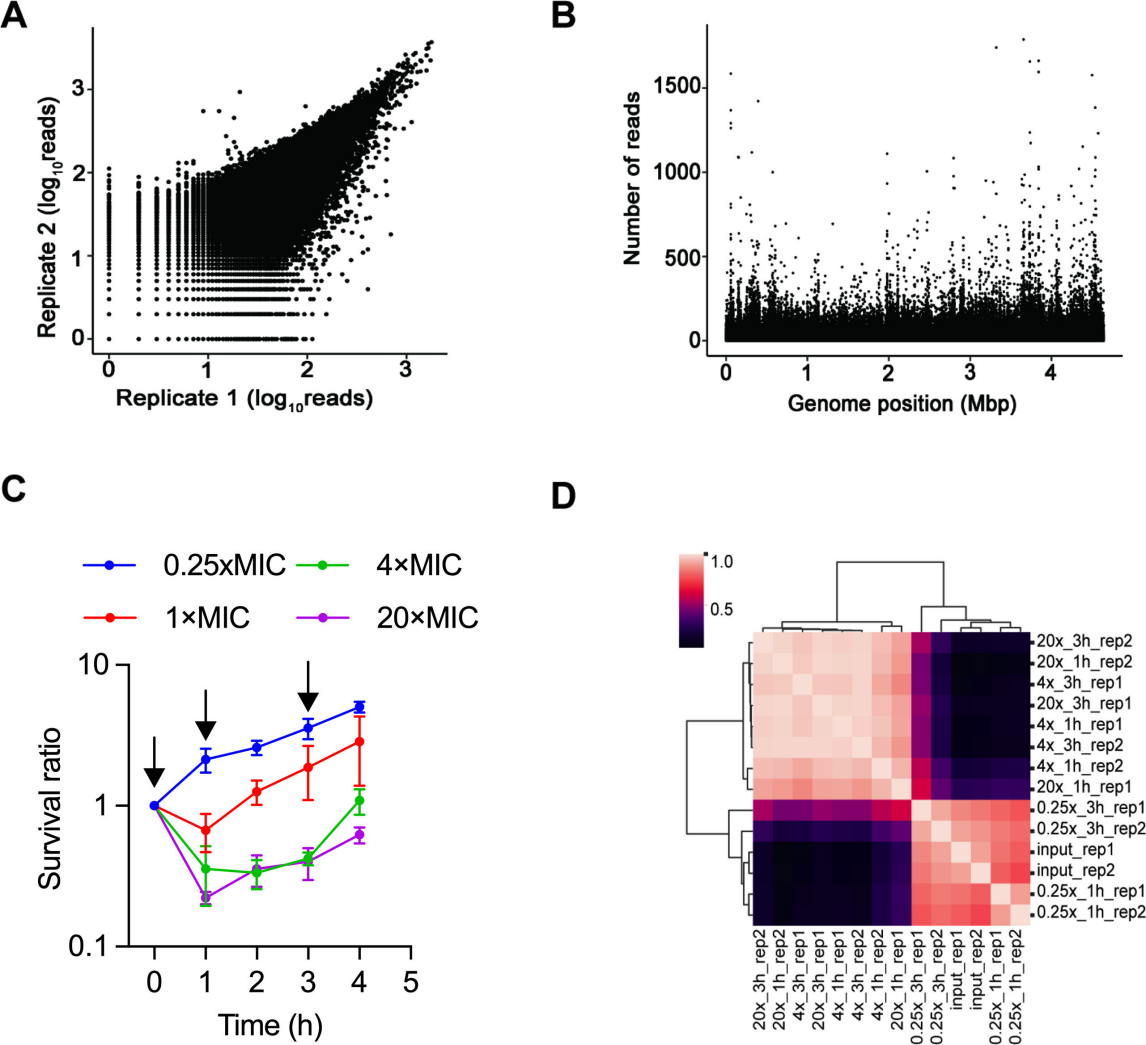
Quality control of the Tn-Seq and screening strategy. (**A**) Correlation coefficients of the number of reads per insertion site for two biological replicates of the input transposon library. Shown are log_10_(reads number +1). (**B**) Distribution of transposon sequence reads across the *E. coli* MG1655 genome. Tn-Seq read data from the input transposon library are shown. (**C**) Survival of the pooled library exposed to rifampicin at the concentrations of 0.25×, 1×, 4×, and 20× MIC. Survival was determined by monitoring colony-forming units (CFUs) and expressed as the ratio compared with pre-treatment. Data are shown as the mean ± SE of three independent experiments. Samples collected before treatment and at 1 and 3 h post-treatment were subjected to Tn-Seq (arrows). (**D**) The cluster heatmap shows Pearson correlation coefficient matrices that measure the relevance of the corresponding samples.

A genome-wide screen was deployed to comprehensively identify the cellular functions involved in modulating the efficacy of rifampicin in the model organism, *E. coli* K12 strain MG1655. The Tn5 transposon insertion mutant library was constructed as previously described (26). Genomic DNA from two biological replicates was subjected to Tn-Seq. The normalized read count of the two biological repeats exhibited a Pearson correlation coefficient of 0.91, demonstrating the high reproducibility of this methodology (Fig. 1A; Table S1). The results showed that this library contained ~310,000 randomly distributed Tn5 insertions, corresponding to an insertion density of 71.4/kb (Fig. 1B).

The *E. coli* K12 strain MG1655 had a rifampicin MIC of 8 mg/L (Fig. S1A). Most bactericidal antibiotics exhibit concentration- and time-dependent killing effects. However, an intriguing characteristic of rifampicin in *E. coli* is the absence of concentration-dependent or time-dependent effects (Fig. 1C). This property indicates that a mutant with a reduced MIC to rifampicin would only be selected by Tn-Seq at subinhibitory concentrations of rifampicin (sub-MIC) but not at concentrations above the MIC (11). Thereafter, to identify the mutants with reduced MIC, rifampicin at 0.25× MIC was selected as a screen condition. In addition, to identify the cellular functions involved in modulating rifampicin efficacy, 4× MIC and 20× MIC were also selected. The pooled library was grown aerobically in the middle exponential phase and exposed to 2 (0.25× MIC), 32 (4× MIC), and 160 (20× MIC) mg/L rifampicin. At 1 and 3 h post-treatment, a sample containing about 3 × 10^6^ viable cells (~10 times the library diversity) was recovered by plating, and genomic DNA was extracted from the pooled outgrowths for Tn-Seq (Fig. 1C). The results of the two biological replicates under each condition were highly reproducible (Pearson correlation coefficient of 0.9 to 0.99) (Fig. 1D).

Next, we used the nonparametric resampling method of the TRANSIT software to identify mutants that were differentially represented between input and post-treatment samples (Table S2) (27, 28). For each gene, the normalized read counts at all the insertion sites and all replicates under each condition were summed. The difference of the summed read counts between the input and post-treatment samples was calculated for each mutant and expressed as a log_2_ fold change (log_2_FC). The significance of this difference was calculated using a permutation test. According to the criteria of log_2_FC > 1 or log_2_FC < −1 and adjusted *P*-value <0.05, a total of 365 genes were selected. Of the identified genes, 216 showed reduced fitness (log_2_FC < −1) upon exposure to rifampicin, suggesting a role in counteracting the action of rifampicin in *E. coli* (Table S3).

### The impact of cellular permeability barriers on rifampicin susceptibility

**Fig 2:**
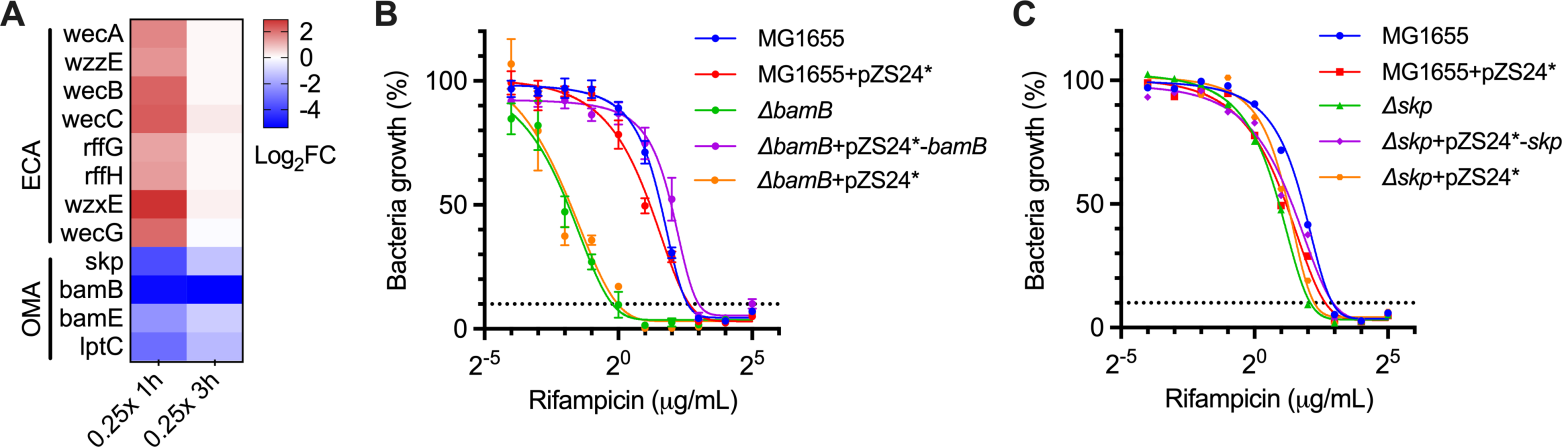
Genes and pathways identified under sub-MIC. (**A**) The heatmap of the log_2_FC values of enriched genes selected under 0.25× MIC. Enriched pathways are labeled on the left: OMA, outer membrane assembly; ECA, biosynthesis of enterobacterial common antigens. (**B and C**) Rifampicin susceptibility (MIC_90_) of *E. coli* MG1655, the mutant strains, and complementary strains harboring the indicated plasmids. The line represents the nonlinear regression of growth that is dependent on the concentration of rifampicin. Data are shown as the mean ± SE of at least three independent experiments.

In accordance with the speculation that the absence of a concentration-dependent killing effect may lead to the selection of mutants with distinct fitness changes between sub-MIC and above-MIC concentrations of rifampicin, we identified 58 genes showing altered fitness exclusively under 0.25× MIC rifampicin (Table S3). Functional categories related to outer membrane assembly (OMA, false discovery rate [FDR] = 0.08) and biosynthesis of enterobacterial common antigen (ECA, FDR = 4.5 × 10^−8^) were significantly enriched among these genes (Fig. 2A; Table S4). OMA is a critical process that maintains the integrity of OM (29), a defining feature of gram-negative bacteria that poses a selective permeability barrier that has evolved to restrict toxic compounds from entering the cell (30). All the selected OMA genes, including *bamB, bamE*, *skp,* and *lptC*, showed reduced fitness upon Tn5 insertion (log_2_FC −2.3 to −5.2). BamB and BamE belong to the β-barrel assembly machinery (BAM complex, BamA–E), responsible for the assembly and insertion of outer membrane proteins (OMPs). Skp is a periplasmic chaperone that functions to remove β-barrel OMPs that have stalled during assembly on the BAM complex (30). To validate these results, we constructed *ΔbamB* and *Δskp* mutants in *E. coli* and found that the inactivation of these genes resulted in eight- and twofold reductions in MIC, respectively (Fig. 2B and C). The increased rifampicin susceptibility of these mutants was completely restored by the expression of *bamB* or *skp* (Fig. 2B and C). Therefore, these results indicate that the BAM complex plays a crucial role in intrinsic resistance to rifampicin.

ECA is a surface carbohydrate antigen composed of repeating units of trisaccharide, which limits the accessibility of hydrophobic compounds to OM (31). However, our results showed that all the selected mutants with Tn5 insertion in the ECA synthesis genes resulted in increased fitness (log_2_FC 1.3 to 2.9), suggesting that ECA may not function as a barrier for rifampicin uptake (Fig. 2A). Similarly, a large-scale chemical genetic screen also showed that deletion of *rffG, rffH, or wecA* in *E. coli* K12 led to reduced sensitivity to sub-MIC rifampicin (32).

### Genes selected under above-MIC concentrations of rifampicin

**Fig 3:**
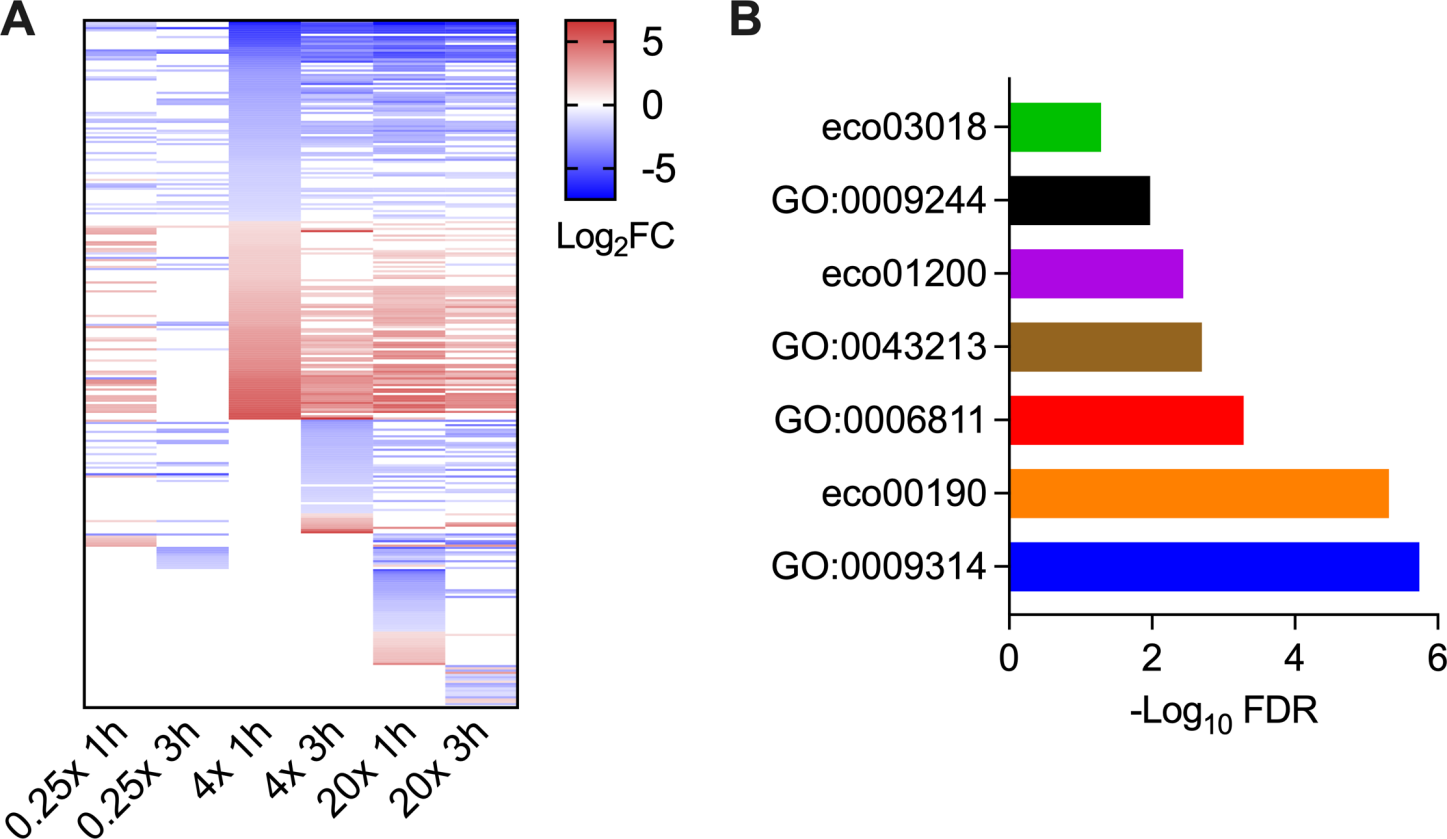
Genes and pathways identified under bactericidal concentrations of rifampicin. (**A**) Heatmap of log_2_FC values of the identified genes. Genes not identified in the condition are shown in white. (**B**) Pathways enriched under bactericidal concentrations of rifampicin. eco03018 (RNA degradation), GO:0009244 (LPS core region biosynthetic process), eco01200 (carbon metabolism), GO:0043213 (bacteriocin transport), GO:0006811 (ion transport), eco00190 (oxidative phosphorylation), and GO:0009314 (response to radiation).

A total of 307 genes were selected under 4× and 20× MIC upon Tn5 insertion (Fig. 3A; Table S3). Among these genes, 115 also exhibited altered fitness under sub-MIC, whereas the others were selected specifically under above-MIC concentrations of rifampicin. By comparing each gene’s fitness change between 4× and 20× MIC, we did not observe apparent drug concentration-dependent killing effect in any of these mutants (Fig. 3A; Table S3). Functional categories related to ion transport (GO:0006811, FDR = 5.2 × 10^−4^), bacteriocin transport (GO:0043213, FDR = 2 × 10^−3^), oxidative phosphorylation (eco00190, FDR = 4.8 × 10^−6^), response to radiation (GO:0009314, FDR = 1.8 × 10^−6^), carbon metabolism (eco01200, FDR = 3.6 × 10^−3^), LPS core region biosynthetic process (GO:0009244, FDR = 0.01), and RNA degradation (eco03018, FDR = 0.051) were significantly enriched among the identified genes (Fig. 3B; Tables S3 and S4).

While most of the enriched genes showed consistent changes in susceptibility to different drug concentrations, three genes (*waaPG* and *galU*) belonging to LPS core region biosynthesis showed opposing fitness changes between sub- and above-MIC concentrations of rifampicin (Table S4). Moreover, although there were seven selected genes (*waaBCDEFOQ*) belonging to the LPS core region biosynthetic pathway that showed consistent changes in susceptibility to different drug concentrations (33), the effects were opposite between *waaBOQ* (increased fitness change) and *waaCDEF* (decreased fitness change) (Table S4). These results indicate that the role of LPS in rifampicin susceptibility may not be akin to its function as a permeability barrier.

### Iron acquisition modulates rifampicin lethality in a hydroxyl radical-independent manner

**Fig 4:**
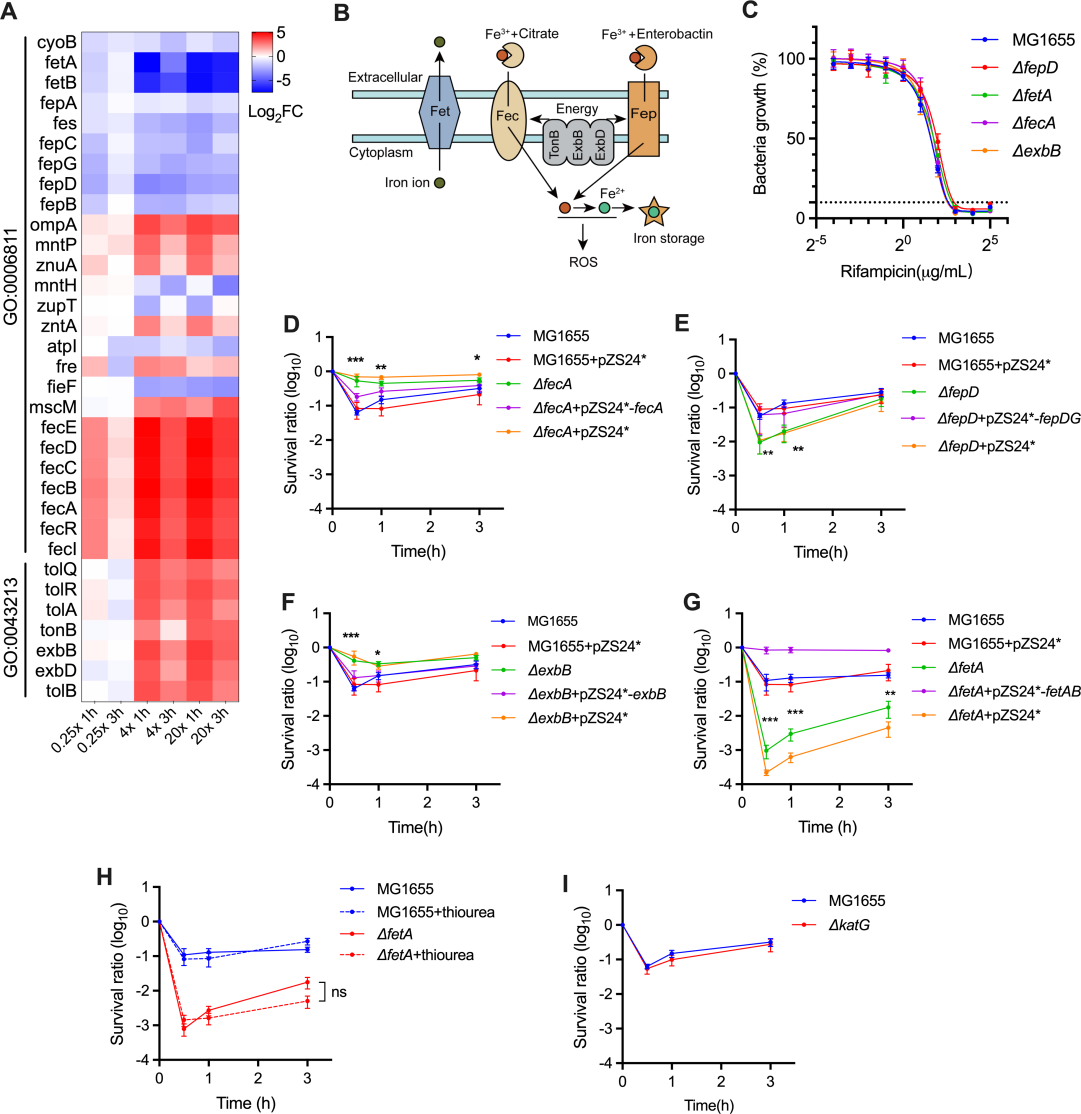
Iron metabolism modulates rifampicin lethality in a hydroxyl radical-independent manner. (**A**) Heatmap of the selected genes involved in iron metabolism. GO:0006811, ion transport; GO:0043213, bacteriocin transport. (**B**) Illustration of the *E. coli* Fe acquisition system selected in our screen. The TonB-ExbB-ExbD complex provides energy to Fep and Fec. (**C**) Rifampicin susceptibility (MIC_90_) of *E. coli* strains. (**D through I**) Survival of the indicated *E. coli* strains exposed to 32 mg/L rifamycin with (**H**) or without the addition of 100 mM thiourea. Data are shown as the mean ± SE of at least three independent experiments. *P < 0.05, **P < 0.01, and ***P < 0.001 using an unpaired *t*-test compared to the counterpart of wild type.

Our results showed that the genes involved in iron metabolism exhibited the most marked changes in fitness and enrichment (Fig. 4A; Table S4). *E. coli* encodes multiple systems, including iron chelating, export, import, and sequestration, to maintain iron homeostasis (Fig. 4B) (34). Among these systems, the FetAB iron exporter showed the most profound fitness reduction upon Tn5 insertion (log_2_FC ~−7), suggesting that shrinking the iron pool may contribute to intrinsic tolerance to rifampicin (Fig. 4A and B) (35). Similarly, mutations in either the ferric citrate importer FecABCDE (log_2_FC ~5) or the TonB complex (encoded by *tonB*, *exbB,* and *exbD*), which energizes iron importers, led to increased fitness (Fig. 4A and B) (36). However, one exception was the FepBCDG ferric enterobactin importer, which exhibited reduced fitness upon Tn5 insertion (Fig. 4A). Although an early study showed that strains with Tn5 insertion in *fepD*, *-C*, and *-G* were unable to grow under iron starvation conditions, its impact on intracellular iron levels under normal growth conditions remains unclear (37). To validate these results, *fetA*, *fepD*, *fecA,* and *exbB* were individually deleted in *E. coli*. We found that the deletion of these genes did not affect rifampicin MIC (Fig. 4C), thus excluding its role in rifampicin uptake (38, 39). The survival abilities of these mutants upon rifampicin treatment were highly consistent with the fitness change observed on the screen. The survival phenotypes of these mutants could be completely restored by the expression of the gene or operon in the mutant strain (Fig. 4D through G).

Iron ions can trigger the generation of hydroxyl radicals (OH^.^) via the Fenton reaction, which can contribute to cell death induced by bactericidal antibiotics (40, 41). However, we found that the addition of the hydroxyl radical scavenger thiourea had no significant effect on the killing efficacy of rifampicin in the wild-type and *ΔfetA* strains (Fig. 4H). Hydrogen peroxide (H_2_O_2_) functions as a substrate to induce hydroxyl radical formation via the Fenton reaction (42). Our results showed that the deletion of *katG*, which encodes the primary scavenger of H_2_O_2_ (43), has no impact on the survival of wild-type *E. coli* upon rifampicin treatment (Fig. 4I). Collectively, these results suggest that iron metabolism contributes to rifampicin lethality in a hydroxyl radical-independent manner in *E. coli*.

### Maintenance of DNA replication and transcription-coupled DNA repair protect *E. coli* cells against killing by rifampicin

**Fig 5:**
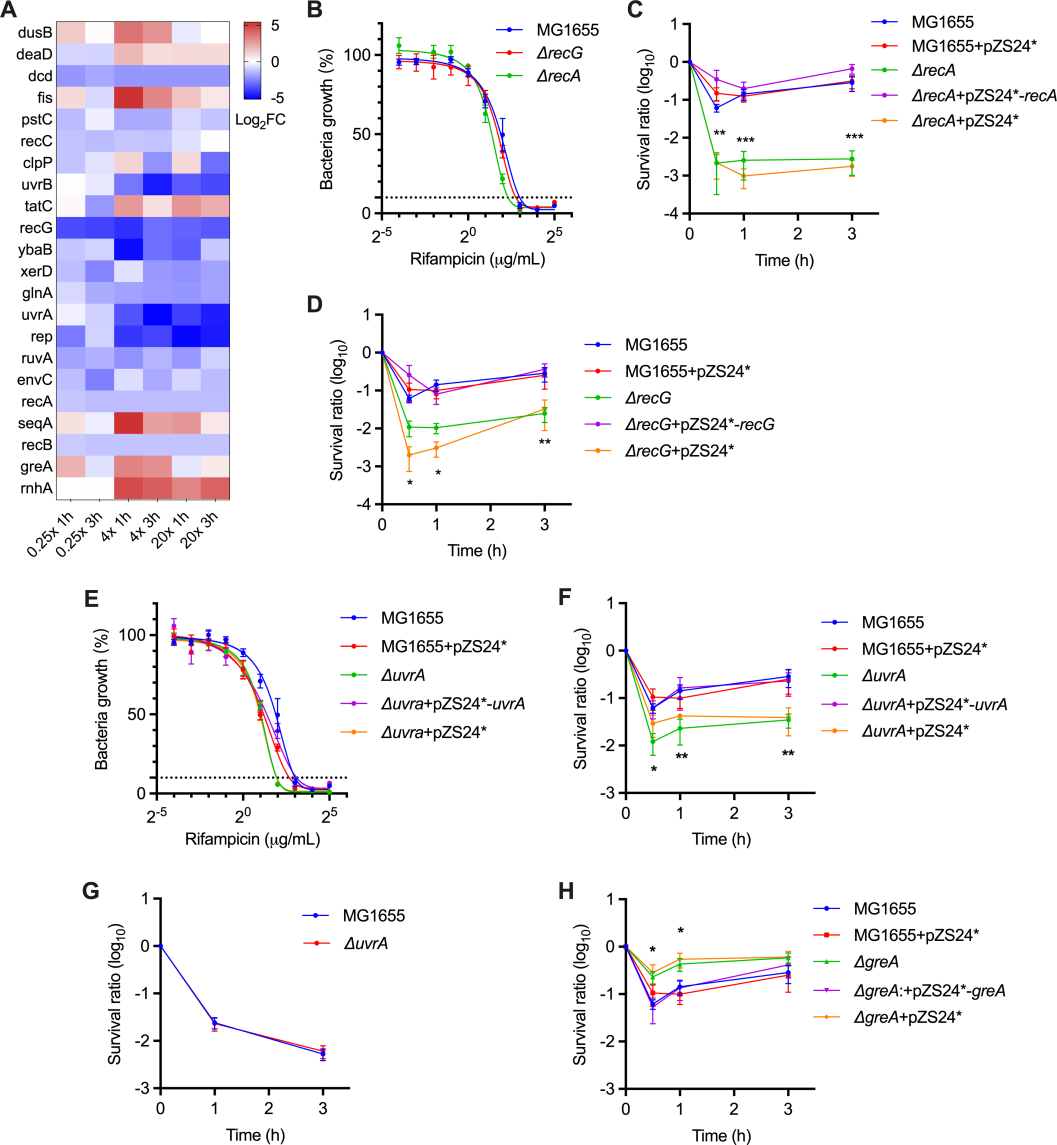
Survival of mutants deficient in DSB-repair function or transcription-coupled DNA repair (TCR) upon exposure to rifampicin. (**A**) Heatmap of the selected genes related to DNA repair and replication. (**B and E**) Rifampicin susceptibility (MIC_90_) of indicated *E. coli* strains. (**C–D and F–H**) Survival of the indicated *E. coli* strains treated with 32 mg/L rifampicin (C, D, F, and H) or 100 mg/L ampicillin (**G**). Data are shown as the mean ± SE of at least three independent experiments. *P < 0.05, **P < 0.01, and ***P < 0.001 using an unpaired *t*-test compared to the counterpart of wild type.

Increasing evidence shows that DNA damage, especially DSBs, is a causative factor for cell death induced by bactericidal antibiotics (11, 17). Although rifampicin is not a typical bactericidal agent in *E. coli*, the reduced fitness of the mutants deficient in recombination repair of DSB (*recABC*, *recG,* and *ruvA*) indicates the engagement of DSB in rifampicin lethality (Fig. 5A) (44). To validate these results, *recA* and *recG* were individually deleted in *E. coli*, and the results showed that the mutants became more sensitive to rifampicin killing without affecting the MIC (Fig. 5B through D). Early studies established that DSBs induced by bactericidal antibiotics could be generated by incomplete base excision repair (BER) (17, 18, 45 – 47). However, our results showed that genes involved in the BER pathway, including the previously validated *mutM*, *mutY*, *mutT, mazG,* and *nth*, were not selected in our screen (Table S4). These results, taken together with the irrelevance of hydroxyl radicals in rifampicin-induced killing (Fig. 4H and I) (5, 24), indicate that rifampicin induces lethal DNA damage in an oxidation-independent manner.

The transcription complex is the primary source of replication fork pausing, which can cause genome instability (48, 49). *E. coli rep* encodes an adenosine triphosphate (ATP)-dependent DNA helicase involved in preventing replication fork stalling by facilitating replication through the transcribed regions (48, 49). The rifampicin-hypersensitive phenotype of *rep* suggests that inhibition of RNAP may cause replication fork pausing, which requires fork bypassing for survival (Fig. 5A). In addition, recent progress has established that the TCR pathway accounts for a vast majority of DNA repair events (50, 51). In this model, UvrA and UvrD associate with RNAP, forming a surveillance complex that can timely backtrack RNAP and initiate nucleotide excision repair (NER) upon encountering DNA lesions during transcription. In accordance with this scenario, our results showed that Tn5 insertion in *uvrA* or *uvrB* resulted in a marked fitness reduction (log_2_FC down to −5.2) among the selected DNA repair genes. Further, we found that the deletion of *uvrA* in *E. coli* not only led to hypersensitivity to rifampicin but also resulted in a twofold reduction in MIC (Fig. 5E and F). These phenotypes were completely restored by the expression of *uvrA* in the mutant strain. Importantly, the deletion of *E. coli uvrA* had no impact on ampicillin killing (Fig. 5G), indicating a unique role of NER in the action of rifampicin. Moreover, the perturbation of TCR upon rifampicin action could also be signified by the increased survival of *greA*-deficient mutant (Fig. 5H). According to recent studies, the inactivation of *greA* in *E. coli* could stimulate RecABCD-mediated DNA repair by promoting the RNAP backtrack (52, 53).

### Sustained cellular respiration and catabolism diminish rifampicin efficacy

**Fig 6:**
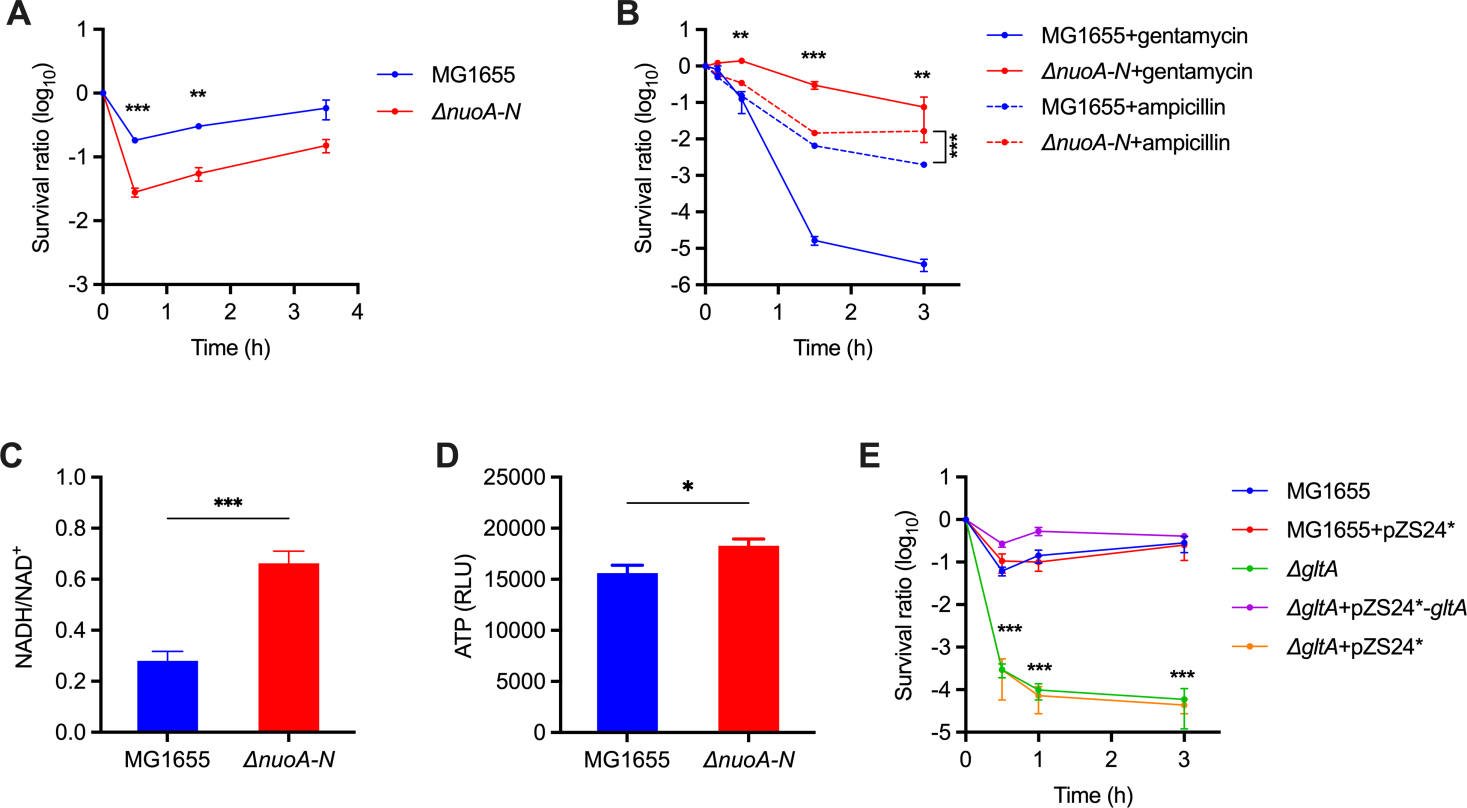
Sustained cellular respiration counteracts rifampicin-induced killing. (**A**) Survival of *E. coli* strains treated with 32 mg/L rifampicin. (**B**) Survival of *E. coli* strains treated with 100 mg/L ampicillin or 16 mg/L gentamycin. (**C and D**) The NADH/NAD^+^ and ATP levels in *E. coli* MG1655 and the *ΔnuoA-N* mutant. RLU, relative light units. (**E**) Survival of *E. coli* strains treated with 32 mg/L rifampicin. Data are shown as the mean ± SE of at least three independent experiments. *P < 0.05, **P < 0.01, and ***P < 0.001 using an unpaired *t*-test compared to the counterpart of wild type.

A previous study established that the lethality of most bactericidal antibiotics is associated with accelerated respiration. However, the action of rifampicin is linked to the inhibition of respiration in various organisms, regardless of its effect on killing (5). The effect of cellular respiration on the lethality of rifampicin remains unclear. Our results showed that genes related to the respiration chain NADH dehydrogenase I (NDH-I, encoded by *nuoA-N*), succinate dehydrogenase (encoded by *sdhA-D*), and *bo*-type oxidase (encoded by *cyoA-D*) were associated with reduced fitness upon Tn5 insertion (Fig. 3B; Tables S3 and S4). Given that deficiency in NDH-I or *bo*-type oxidase leads to significantly reduced oxygen consumption in *E. coli* (54), these results indicate that sustained aerobic respiration may counteract rifamycin killing. To test this hypothesis, we constructed an unmarked *ΔnuoA-N* strain in *E. coli*, and the results showed that the mutant became more sensitive to rifampicin (Fig. 6A). The deletion of *nuoA-N* in *E. coli* resulted in a growth-deficient phenotype in enriched medium (Fig. S1B), consistent with previous findings that NDH-1 predominates when *E. coli* is grown at high oxygen tension (54, 55). The deletion of *nuoA-N* in *E. coli* did not affect the MIC of rifampicin (Fig. S1A), suggesting a role of NDH-1 in rifampicin’s killing, rather than drug entry. Moreover, our results indicated that the NDH-1-added survival effect specifically relies on the inhibition of transcription by rifampicin, and the deletion of *nuoA-N* in *E. coli* resulted in decreased killing by ampicillin or gentamycin and did not affect the sensitivity to spectinomycin, chloramphenicol, and tetracycline (Fig. 6B; Fig. S1C) (5). Collectively, these results suggest a role for sustained aerobic respiration in counteracting rifampicin-induced killing.

Aerobic respiration plays a pivotal role in cell metabolism by recycling NAD^+^ and generating ATP. To dissect the mechanisms underlying NDH-I-induced survival, we measured ATP and NADH/NAD^+^ in the *ΔnuoA-N* strain. Our results showed that NDH-I deficiency resulted in a greater than twofold increase in the NADH/NAD^+^ ratio, whereas its effect on cellular ATP was very mild (Fig. 6C and D). Given that the regeneration of reducing equivalents is critical for the maintenance of cellular catabolism, these results indicate that the effect of NDH-I depletion on rifampicin killing may be attributable to reduced catabolism (56). This speculation could be supported by the selection of genes enriched in carbon metabolism (eco01200, FDR = 3.6 × 10^−3^) (Fig. 3B; Table S4). These metabolic genes mainly belonged to the tricarboxylic acid (TCA) cycle (including *gltA*, *sdhB*, *mdh,* and *fumC*), pentose phosphate pathway (*tpiA*, *talB*, *tktA*, *rpiA*, *rpe*, and *gnd*), and glycolysis (*pgi*, *pfkA,* and *pykF*), almost all of which exhibited reduced fitness upon Tn5 insertion (except for *gnd* encoding for 6-phosphogluconate dehydrogenase). To validate our hypothesis further, we constructed a mutant deficient in *gltA,* which encodes the first committed enzyme of the TCA cycle. The results showed that, upon exposure to rifampicin, the survival rate of the *ΔgltA* mutant was reduced by three orders of magnitude compared to the wild type (Fig. 6E). This phenotype was completely restored by *gltA* expression in the mutant strain. These results provide evidence that sustained NAD^+^ recycling and carbon catabolism counteract the killing effect of rifampicin.

## DISCUSSION

Rifampicin plays a pivotal role in shortening the course of antituberculosis therapy owing to its sterilizing activity against *M. tuberculosis* (4). However, owing to its low bactericidal activity and rapid development of resistance, its clinical application in infectious diseases caused by most gram-negative bacteria is limited. In this study, we comprehensively identified and evaluated the cellular functions that modulate the action of rifampicin in the model organism *E. coli* and provided unique insights into its lethal effects.

Our results showed that *E. coli* mutants deficient in DSB-repair genes *recA-C*, *recG,* and *ruvA* became more sensitive to rifampicin killing, suggesting a role of lethal DNA damage in rifampicin killing (6). Although the precise mechanisms underlying rifampicin-induced DNA damage remain elusive, our study revealed the unique characteristics of its action. First, in accordance with the inability of rifampicin to induce ROS in *E. coli* (5, 24), we found that the well-characterized BER genes that contribute to ROS-induced DSB were not selected upon rifampicin action (17, 18, 45 – 47). These findings, taken together with the results showing the irrelevance of *katG* and thiourea in rifampicin’s killing, signify distinct oxidation-independent DNA damage events. Further, we provided genetic evidence that rifampicin-induced DNA damage may stem from perturbations in DNA replication and TCR. The implication of Rep in rifampicin lethality suggests that the inhibition of RNAP may result in a stalled transcriptional complex, leading to replication fork collision and pausing (48, 49). The participation of TCR in rifampicin’s action could be exemplified by the hypersensitivity of the mutants deficient in UvrA/UvrB, as well as the protective effect posed by GreA depletion. In *E. coli*, UvrA, UvrD, and RNAP form a surveillance complex that can timely backtrack RNAP and initiate NER upon encounter with a DNA lesion during transcription (50, 51). By contrast, the presence of GreA impedes RNAP backtracking (52, 53). Our results demonstrated that the deletion of *uvrA* in *E. coli* resulted in a twofold reduction in rifampicin MIC but did not affect ampicillin sensitivity, suggesting a unique role of NER in rifampicin action.

Early studies have established the role of the ferrichrome transport system (FhuA-TonB) encoded by some gram-negative bacteria in the uptake of rifamycin derivatives CGP 4832 and rifabutin, but not of rifampicin (38, 39, 57). While our results are consistent with these findings, showing the unchanged rifampicin MIC of the mutant deficient in the TonB complex and other iron transporters, it also suggests a novel role of iron metabolism in rifamycin killing. Moreover, the evidence presented in this study suggests that the impact of iron on rifampicin’s killing does not rely on the generation of hydroxyl radicals, which is strikingly different from the well-established role of iron in ROS-mediated cell death (40, 41). Together, these observations suggest an alternative route for iron-related cell death. Notably, antibiotic lethality under anaerobic conditions can be induced by reactive metabolic byproducts in *E. coli* (58). Considering the inhibitory effect of rifampicin on cellular respiration (5), further studies should address the physiological role of iron under oxygen-limited conditions.

For most bactericidal antibiotics, the inhibition of bacterial respiration counteracts killing (5). However, our results indicate that reduced aerobic respiration contributes to rifampicin-induced killing of *E. coli*. We found that the depletion of NDH-I in *E. coli* resulted in increased killing by rifampicin, which could be attributed to increased NADH/NAD^+^ and reduced carbon catabolism. Moreover, our results demonstrated that this effect relies on the inhibition of transcription but not translation. It is possible that the reduced but sustained respiration and catabolism add to survival by providing building blocks for the repair of cellular damage induced by rifampicin.

Penetration through the OM is the rate-limiting step in the action of rifampicin (1). In *Enterobacterales*, LPS and ECA are the key surface components that limit the accessibility of the hydrophobic compounds to OM (30, 31, 33). Integral OMPs also play a role in OM permeability owing to the selective transport of small hydrophilic molecules (29, 30). Our results showed that the BAM complex contributes substantially to the impermeability of rifampicin. However, we found that mutants deficient in ECA biosynthesis universally exhibited increased fitness under sub-MIC rifampicin, suggesting that ECA may not function as a barrier to rifampicin entry (32). Intriguingly, our results indicated that the impact of LPS on rifampicin efficacy is mild and may have opposing effects between sub-MIC and above-MIC concentrations of rifampicin. These results suggest that the contribution of these permeability barriers to rifampicin entry differs substantially.

Our findings provide evidence that bacterial physiological and metabolic responses may account for the discrepancy in the efficacy of rifamycins in different organisms. For instance, in *Mycobacterium*, rifampicin’s lethality has been demonstrated to be dependent on the generation of ROS and oxidative DNA damage (17, 20 – 23). Moreover, a recent study showed that mycobacteria deploy increased iron acquisition to counteract oxidative damage, which is quite distinct from the defense tactics of *E. coli*, characterized by shrinking of the iron pool (23). Therefore, a deeper understanding of these physiological and metabolic responses in the context of rifamycin efficacy may aid in the development of new therapeutic strategies and antimicrobial adjuvants.

## MATERIALS AND METHODS

### Strains, media, and growth conditions

*E. coli* K12 strain MG1655 was used in this study. *E. coli* was cultured in Luria–Bertani (LB) and LB agar media. Antibiotics at indicated concentrations were added to the medium. Cells were grown at 37°C on a rotating shaker at 220 rpm.

### Generation of transposon mutant library

A transposon mutant library was generated in *E. coli* following the method described for *Salmonella typhi* (26). Briefly, a modified Tn5 transposon (Epicentre, TSM99K2) was transformed into *E. coli* by electroporation. Transformants were grown on LB agar supplemented with kanamycin (50 mg/L); the kanamycin-resistant colonies were pooled in LB containing 12% glycerol and stored at −80°C.

### Library screening

The transposon mutant library was grown in 10 mL LB supplemented with kanamycin (50 mg/L) in a 50-mL flask at 37°C to an OD_600_ of ~1.0. Cells were washed with an equal volume of LB to remove kanamycin. Approximately 10^7^ colony-forming units were inoculated into a 100-mL flask containing 10-mL LB and grown to an OD_600_ of 0.5. Input samples were collected at this stage by centrifugation. The culture was then aliquoted to three flasks and treated with different concentrations of rifampicin (2, 32, and 160 mg/L, Sigma Aldrich, R3501) at 37°C with rotation. At 1 and 3 h post-treatment, approximately 3 million CFUs were recovered by plating onto five LB agar plates (200 mm diameter) supplemented with kanamycin (50 mg/L) and incubated overnight at 37°C. Colonies were pooled in LB and stored at −80°C. Two biological replicates were performed.

### DNA preparation and Tn-Seq

Harvested cells were prepared for Tn-Seq as previously described (26, 59). Briefly, genomic DNA was extracted from cell pellets using the TIANamp bacteria DNA kit (Tiangen). DNA was quantified and mechanically sheared by ultrasonication. Sheared DNA fragments were subjected to end repair, dA-tailing, ligation with Illumina Truseq adaptors, and PCR using an adaptor and Tn5-specific primers. After PCR purification, Illumina-specific flow adaptor sequences were added through an additional PCR before sequencing. Adaptors and primers used in this study are listed in Table S5.

### Sequencing analysis

Data analysis was performed using TPP and TRANSIT (27). Raw data were processed using TPP and BWA (60). Raw sequencing reads were first trimmed using sequence 5′-GAGATGTGTATAAGAGACAG-3′ to remove the transposon sequence; reads with a flanking genome region longer than 20 bp were retained. Then, trimmed reads with proper indexes were mapped to a unique site on the *E. coli* MG1655 genome (NCBI GenBank, NC_000913.3). Finally, mapped reads were filtered by their indexes to eliminate PCR bias. Those insertion sites in the genome and the count of each read were recorded in a single wig file. According to the file, Pearson correlation and distribution of reads can be calculated and visualized with Python and R. Fitness change was determined by carrying out a pairwise comparison of each rifampicin-treated sample with the input data. Reads in the 5% N-terminal and 5% C-terminal of the gene sequence were discarded (61). The “Resampling” method of TRANSIT software (v3.2.0) was used to identify mutants that were differentially represented between input and post-treatment samples. Read counts, *P*-value (adjusted by using the method of FDR), and log_2_FC between the input and post-treatment were calculated using default parameters. The genes with an adj *P*-value of less than 0.05 and log_2_FC of more than 1 or less than −1 were selected.

### Gene enrichment analysis

Pathway enrichment analysis was performed on the website (https://david.ncifcrf.gov). Enrichment in pathway terms was calculated for each gene cluster. GO terms with an FDR value of less than 0.1 were selected.

### Gene deletion and complementation

Single gene deletion in *E. coli* was constructed by allelic transduction from Keio collection strains using classical P1 phage transduction followed by Kan^R^ cassette excision (62). For the deletion of *nuoA-N*, DNA fragments containing flanking regions of the *nuoA-N* cluster and Kan^R^ cassette were PCR-amplified and transformed into *E. coli* using electroporation. Recovered cells were selected for kanamycin-resistant homologous recombinants. To avoid potential off-target effects, the selected mutant containing the Δ*nuoA-N*::Kan^R^ allele was used as a donor for P1 transduction to generate the Δ*nuoA-N* mutant. The plasmid was cured, and the Kan^R^ cassette was removed to generate the unmarked *nuoA-N* deletion mutant. The mutant genotype was verified by PCR and sequencing. For complementation of the gene deletion mutants, the coding region of the target gene was PCR-amplified and cloned into a low-copy plasmid pZS*24 (63). The resulting plasmid was transformed into the mutant strain by electroporation.

### Antibiotic susceptibility

To measure MIC, strains were grown to the exponential phase and diluted to ~10^5^ CFU per mL in 200-µL LB media containing antibiotics at indicated concentrations. Growth was monitored by OD_600_ and expressed as percent of growth relative to the untreated control. Ninety percent inhibitory concentration (MIC_90_) was determined by plotting the percent of growth versus concentration of antibiotic. The growth inhibition dynamics were approximated by nonlinear regression using the inhibitor versus response model of GraphPad Prism 9.0. Three biological replicates were performed.

### Antibiotic killing assay

Bacterial cultures were grown overnight in LB and then diluted 1:200 in 10-mL LB in 50-mL flasks. For the complementary strains, cultures were grown in LB containing 20 µg/mL kanamycin; gene expression was induced by 1 mM isopropyl-1-thio-β-D-galactopyranoside upon inoculation of the seeding culture. At an OD_600_ of 0.3 to 0.4, aliquots of 1 mL culture (in a 14-mL tube) were treated with antibiotics at 37°C. Aliquots were taken, serially diluted, and plated onto LB agar plates to determine CFU. Survival was expressed as the ratio compared with pre-treatment.

### ATP qualification

ATP concentration was measured by using the BacTiter-Glo Microbial Cell Viability Assay kit (Promega) and FlexStation 3 (Molecular Devices) following the manufacturer’s protocols. Samples (100 µL) at an OD_600_ of 0.4 to 0.6 were mixed with an equal volume of BacTiter-Glo reagent in 96-well opaque plates and incubated at room temperature for 5 minutes. The relative light units were recorded by FlexStation 3 and normalized by OD_600_.

### NADH/NAD^+^ assay

The qualification of NADH and NAD^+^ was performed by using the NAD/NADH-Glo Assay kit (Promega) and FlexStation 3 following the manufacturer’s protocols. The samples (100 µL) at an OD_600_ of 0.4 to 0.6 were mixed with an equal volume of NAD/NADH-Glo detection reagent in 96-well opaque plates and incubated at room temperature with gentle shaking for 60 minutes. RLU values were recorded by FlexStation 3 and normalized by OD_600_.

### Statistical analysis

Statistical analysis was performed on log_10_-transformed data (for antibiotic killing experiments) or untransformed data (for NADH and ATP assays) using unpaired two-tailed Student’s *t*-test using GraphPad Prism 9.0 software. A statistical difference between wild type and a gene deletion mutant is marked (*P < 0.05; **P < 0.01; ***P < 0.001).

## SUPPLEMENTAL MATERIAL

Fig.S1, Tables S1 and S5 spectrum.02895-23-s0001.pdf Supplemental material.

Table S2 spectrum.02895-23-s0002.xlsx Raw data of resampling analysis.

Table S3 spectrum.02895-23-s0003.xlsx Identified genes.

Table S4 spectrum.02895-23-s0004.xlsx Pathway enrichment analysis.

ASM does not own the copyrights to Supplemental Material that may be linked to, or accessed through, an article. The authors have granted ASM a non-exclusive, world-wide license to publish the Supplemental Material files. Please contact the corresponding author directly for reuse.
