# Supplementary material for: Genome-wide screen reveals cellular functions that counteract rifampicin lethality in Escherichia coli
Source: Microbiol Spectr. 2023 Dec 6;12(1):e02895-23. doi: 10.1128/spectrum.02895-23 (PMC10782999; doi:10.1128/spectrum.02895-23)
Supplement: Fig.S1, Tables S1 and S5 — Supplemental material. [file spectrum.02895-23-s0001.pdf]

Table S1. Statistics of the Tn-seq data

Sample	Total reads	Mapped reads	Template reads	insertion sites
input rep1	25,753,217	24,404,314	6,500,135	310,306
input rep2	31,753,300	30,205,098	8,836,837	315,462
0.25X_1h_rep1	21,380,597	20,315,792	7,043,278	264,992
0.25X_1h_rep2	19,447,306	18,334,126	6,297,947	231,769
4X_1h_rep1	20,757,959	19,441,561	6,237,121	169,843
4X_1h_rep2	32,511,060	31,375,440	8,412,891	173,228
20X_1h_rep1	26,169,545	25,181,922	8,804,567	198,803
20X_1h_rep2	30,306,167	29,298,859	8,421,783	171,766
0.25X_3h_rep1	19,972,476	19,242,890	8,302,520	277,957
0.25X_3h_rep2	22,424,442	21,589,488	6,146,798	256,845
4X_3h_rep1	26,113,643	24,981,615	8,229,931	233,279
4X_3h_rep2	25,581,091	24,660,957	7,248,518	199,745
20X_3h_rep1	28,846,007	27,221,795	9,050,211	213,391
20X_3h_rep2	29,543,869	28,362,082	5,477,620	177,598

Table S5. Primers used in this study

Primer	Sequence (5'->3')	Description
Tn-adaptor-F	ATGATGGCCGGTGGATTTGTGNNANNANNNTGGTCGTGGTAT	ligate with genome
Tn-adaptor-R	TACCACGACCA-NH2	DNA fragments;
Tn-PCR1-F	ATGATGGCCGGTGGATTTGTG	enrich DNA fragments
Tn-PCR1-R	AAAGCTCTCATCAACCGTGG	containing transposon;
Tn-PCR2-F	AATGATACGGCGACCACCGAGATCTACACTCTTTCCCTACACGACGCTCTTCCGATCTCG	bridge amplification
	GGGATCCTCTAGAGTCGACCTGC	before sequencing;
Tn-PCR2-R	CAAGCAGAAGACGGCATACGAGATTGTTTCGAGTGACTGGAGTTCAGACGTGTGCTCTTC	
	CGATCTATGATGGCCGGTGGATTTGTG	
nuo-KO-F	GAGCAGTGAATCTGGCGCTACTTTTGATGAGTAAGCAATGATTCCGGGGATCCGTCGA	knock out *nuoA-N*
nuo-KO-R	TGACTTACAAAGTAACAGATTACATCAGCGGCATTGCCAATGTAGGCTGGAGCTGCTT	
nuo-test-F	GTCGTCAAGGATCTGGGGTG	verify *ΔnuoA-N*
nuo-test-R	AGAGAAAACCGCTTCAGGCA	
bamB-KO-F	GAAAATTAATAATTTGTCCATCTGAGAGGGACCCGATGATTCCGGGGATCCGTCGA	knock out *bamB*
bamB-KO-R	CCAGGAGCCGTTTTCAAAGTGAACGACAGAGACGATTATGTAGGCTGGAGCTGCTT	
bamB-test-F	CCTGCGTGGTGAAGCATTGC	verify *ΔbamB*
bamB-test- R	TAGCAGCCAGTCCCTTCCCG	
skp-test-F	CTCCTACGCCCAGCCGTTC	verify *Δskp*
skp-test-R	GGGTCATGACAACCGCGGAC	
recG-test-F	GCCGGAAGCAGAGCAACAAC	verify *ΔrecG*
recG-test-R	CTTTGGCCCGTCGCACATG	
recA-test-F	GGGAATGCTTCAGCGGCGAC	verify *ΔrecA*
recA-test-R	CCATAATCGGTGCCGCGAG	
fecA-test-F	CGGGACGTTCCCGCTGAAA	verify *ΔfecA*
fecA-test-R	CACCACAATCCGTTGTGGCG	
fepD-test-F	TCATCTGGATCTGCACCGGC	verify *ΔfepD*
fepD-test-R	GCGGGTTACGCATCAGCGAC	
fetA-test-F	CCGACAATGACCAGCGCGAC	verify *ΔfetA*
fetA-test-R	GGCTGGCATCATCCACGCTG	
katG-test-F	CTGCCTCGAAATGAGGGCGG	verify *ΔkatG*
katG-test-R	AGTGTGAGCACAACCAGGCC	
exbB-test-F	GCAAATGGCGTGCTGGTGAC	verify *ΔexbB*
exbB-test-R	CGGCGTGCTGGTAGAAGCAG	
greA-test-F	CGCTGCCACGCTAAGATTATCC	verify *ΔgreA*
greA-test-R	CTGACCTGGGACTTGTACCCGG	
uvrA-test-F	GCACCGTGTATCTCGACGGTTC	verify *ΔuvrA*
uvrA-test-R	CGGGAAACAAACCTGGCCAGAC	
gltA-test-F	GATACGGCCAGTGCGGCCAATG	verify *ΔgltA*
gltA-test-R	CTGGGTGGCTCGGGATTGCAGGG	

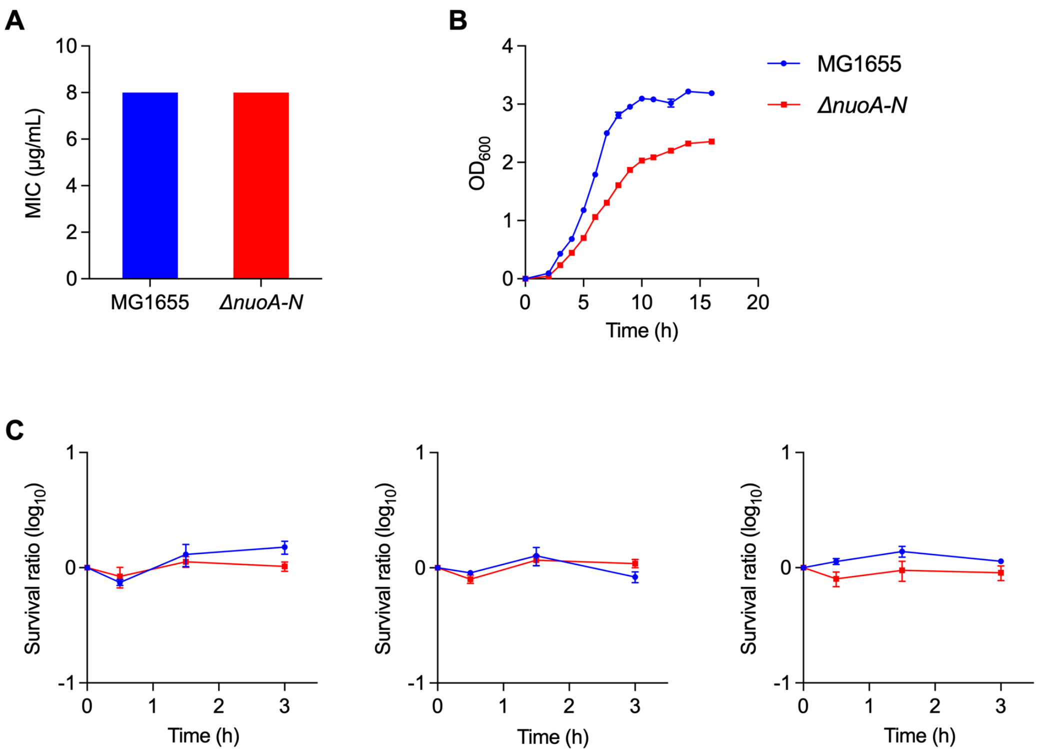

Figures S1. (**A**) Rifampicin susceptibility (MIC$_{90}$) of *E. coli* MG1655 and the *ΔnuoA-N* mutant. (**B**) Grow curves of *E. coli* MG1655 and the *ΔnuoA-N* mutant. (**C**) Survival of *E. coli* MG1655 and the *ΔnuoA-N* mutant treated with spectinomycin (64 μg/ml, left), chloramphenicol (50 μg/ml, middle), tetracycline (10 μg/ml, right). Data shown are mean ± SE in triplicate.
